# Role of Endothelium in Cardiovascular Sequelae of Long COVID

**DOI:** 10.3390/biomedicines11082239

**Published:** 2023-08-09

**Authors:** Luca Santoro, Vincenzo Zaccone, Lorenzo Falsetti, Vittorio Ruggieri, Martina Danese, Chiara Miro, Angela Di Giorgio, Antonio Nesci, Alessia D’Alessandro, Gianluca Moroncini, Angelo Santoliquido

**Affiliations:** 1 Department of Cardiovascular and Thoracic Sciences, Fondazione Policlinico Universitario Agostino Gemelli IRCCS, 00168 Rome, Italy; luca.santoro@policlinicogemelli.it (L.S.); angela.digiorgio@policlinicogemelli.it (A.D.G.); antonio.nesci@policlinicogemelli.it (A.N.); alessia.dalessandro@guest.policlinicogemelli.it (A.D.); angelo.santoliquido@policlinicogemelli.it (A.S.); 2 Department of Emergency Medicine, Internal and Sub-Intensive Medicine, Azienda Ospedaliero-Universitaria delle Marche, 60126 Ancona, Italy; 3 Clinica Medica, Department of Clinical and Molecular Sciences, Università Politecnica delle Marche, 60126 Ancona, Italy; drfalsetti@yahoo.it (L.F.); g.moroncini@staff.univpm.it (G.M.); 4 Department of Internal Medicine, Università Cattolica del Sacro Cuore, 00168 Rome, Italy; vit_ruggieri@yahoo.it (V.R.); martinadanese96@yahoo.it (M.D.); chiaramiro23@gmail.com (C.M.); 5 Università Cattolica del Sacro Cuore, 00168 Rome, Italy

**Keywords:** long COVID, post-COVID-19, post-acute sequelae SARS-CoV-2 infection (*PASC*), SARS-CoV-2 infection, endothelium, cardiovascular disease

## Abstract

The global action against coronavirus disease 2019 (COVID-19), caused by SARS-CoV-2 infection, shed light on endothelial dysfunction. Although SARS-CoV-2 primarily affects the pulmonary system, multiple studies have documented pan-vascular involvement in COVID-19. The virus is able to penetrate the endothelial barrier, damaging it directly or indirectly and causing endotheliitis and multi-organ injury. Several mechanisms cooperate to development of endothelial dysfunction, including endothelial cell injury and pyroptosis, hyperinflammation and cytokine storm syndrome, oxidative stress and reduced nitric oxide bioavailability, glycocalyx disruption, hypercoagulability, and thrombosis. After acute-phase infection, some patients reported signs and symptoms of a systemic disorder known as long COVID, in which a broad range of cardiovascular (CV) disorders emerged. To date, the exact pathophysiology of long COVID remains unclear: in addition to the persistence of acute-phase infection mechanisms, specific pathways of CV damage have been postulated, such as persistent viral reservoirs in the heart or an autoimmune response to cardiac antigens through molecular mimicry. The aim of this review is to provide an overview of the main molecular patterns of enduring endothelial activation following SARS-CoV-2 infection and to offer the latest summary of CV complications in long COVID.

## 1. Introduction

Coronavirus disease-19 (COVID-19) was first reported on 8 December 2019 in Hubei province in China, but the epicenter shifted from China to Europe and the rest of the world starting from February/March 2020 [1]. It is primarily a respiratory infection caused by the severe acute respiratory syndrome coronavirus 2 (SARS-CoV-2). Nonetheless, it may involve multiple other organs, including the cardiovascular (CV) system, with several possible complications, such as pulmonary embolism, deep vein thrombosis, stroke, acute myocardial infarction, heart failure, myocarditis, pericarditis, and arrhythmias [1,2].

SARS-CoV2 interacts with the surface receptor of angiotensin-converting enzyme 2 (ACE2). ACE2 is present in several cells types, such as endothelium, bronchial epithelium, and type-2 alveolar cells [3,4]. The vascular damage is caused both directly by the viral cytopathic effect on endothelial cells and indirectly as a result of endothelial activation due to systemic inflammatory cytokine storm [5,6]. These mechanisms trigger a hypercoagulable inflammatory state characterized by increased vascular permeability, thrombosis, vasoconstriction, reactive oxygen species (ROS) production, and apoptosis/pyroptosis, leading to endotheliitis [2,7]. The outlined COVID-19 endothelial inflammation could explain the systemic impaired microcirculatory function in different vascular beds and the clinical sequelae in patients with acute COVID-19 [6].

Additionally, more and more patients complained of signs and symptoms developing after SARS-CoV-2 infection and persisting for more than 12 weeks, not explained by an alternative diagnosis. This new nosographic entity has been defined as long COVID or post-COVID-19 syndrome by the National Institute for Health and Care Excellence (NICE) [8].

Also related to these subjects, a range of CV abnormalities has been described, including myocardial and pericardial inflammation, myocardial infarction, arrhythmias and thrombo-embolic events [9]. Peculiar mechanisms of CV damage have been postulated in long COVID, such as a chronic inflammatory response evoked by persistent viral reservoirs in the heart following the acute infection or an autoimmune response to cardiac antigens through molecular mimicry [10,11]. This review aims to provide insight into the possible role of the endothelium as the red thread between SARS-CoV-2 infection and CV sequelae in long COVID patients.

## 2. Endothelium and SARS-CoV-2 Infection

The vascular endothelium is the innermost layer of blood vessels and, at the same time, a dynamic structure that is vital for the regulation of vascular health and homeostasis. Endothelial cells have many physiological properties: as a gatekeeper, they firstly provide a natural barrier to blood borne pathogens, recognizing the danger and spreading signals of infection or injury; as a control room, they regulate processes such as vascular tone, hemostasis, inflammation, oxidative stress, and vascular permeability, acting as an autocrine, paracrine, and endocrine organ. In other words, under normal circumstances, the endothelium balances every process, releasing molecules to preserve antioxidant, anti-inflammatory, anti-thrombotic, and anti-proliferative functions [2,12].

Consolidated evidence describes COVID-19 as a pan-vascular disease in which the endothelium has been regarded as its Achilles′ heel [7,12,13,14].

Although SARS-CoV-2 infection primarily affects the pulmonary system, COVID-19 can present as a multi-organ disease because of endothelial dysfunction/endotheliopathy/endotheliitis, in which there is a shifting of the vascular equilibrium towards vasoconstriction, inflammation, and a procoagulant state [15,16,17].

**Figure 1:**
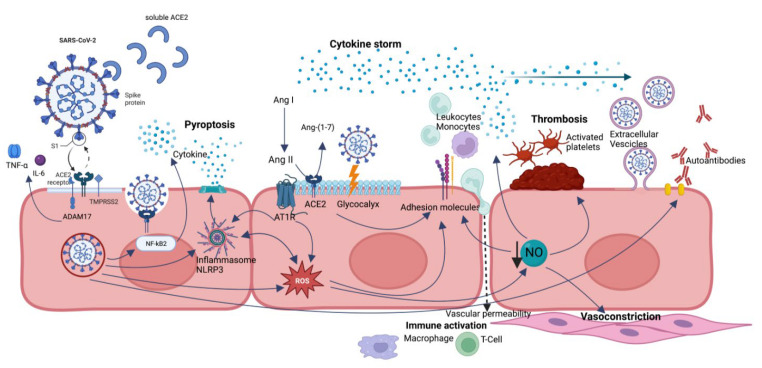
A summary of endothelial dysfunction pathways caused by SARS-CoV-2 infection. Direct viral infection is mediated by ACE2 receptor and ADAM17 and allows SARS-CoV-2 penetration into the cell, processes ACE2 in its soluble form (reducing its protective role), and sheds membrane-anchored cytokines (TNF-alfa and IL-6). ACE2 dysregulation leads to a reduction in Ang-(1-7) and accumulation of Ang II, with imbalance in favor of Ang II/AT1R axis with proinflammatory effects. NLRP3 inflammasome is activated by several mechanisms, in particular direct interaction with viral nucleocapsid (N) protein, hyperactivated AngII-AT1R axis, and ROS, leading to pyroptosis. Oxidative stress results from ROS overproduction and reduced NO bioavailability and contributes to endothelial dysregulation, such as overexpression of adhesion molecules, augmented vascular permeability, platelet adhesion and aggregation, smooth muscle cell proliferation, and vasoconstriction. Glycocalyx disruption also leads to increased vascular permeability and loss of natural barrier function. Extracellular vesicles can transport proinflammatory and pro-thrombotic molecules and even the virus to distant sites. All these effects are mediated not only by direct SARS-CoV-2 infection but also by persistence of soluble S and N proteins.

SARS-CoV-2 can cause endotheliopathy through direct (via virus infection) and indirect (via cytokine storm) mechanisms [6] (Figure 1).

The entry of SARS-CoV-2 into cells is mediated by its obligate receptor, the ACE2 receptor [18]. ACE2 is a type I transmembrane carboxymonopeptidase protein. It is ubiquitously expressed in endothelial cells of several organs, such as the respiratory system (in type II alveolar cells, but especially in the upper bronchial epithelium and, in higher concentration, in the ciliated cells of the nasal epithelium), small intestine, testis, kidney, heart muscle, colon, nervous system (particularly the olfactory bulb), and thyroid gland [19,20,21,22].

SARS-CoV-2 infection is prompted by the efficient binding of the ACE2 receptor with the surface spike (S) protein [23,24]. The infection process is facilitated by the transmembrane protease serine 2 (TMPRSS2) [24]. 

In addition, disintegrin and metalloprotease 17 (ADAM17), also called TACE (tumor necrosis factor-α-converting enzyme), permits penetration of SARS-CoV-2 into cells by proteolytically processing ACE2 and favoring its shedding into the extracellular space, also cleaving and releasing in the extracellular space some membrane-anchored immunological cytokines, such as tumor necrosis factor-α (TNF-α) and interleukin (IL)-6 [25].

The interaction between ACE2 and SARS-CoV-2 determines a disruption of the physiological renin–angiotensin–aldosterone system (RAAS)—which ACE2 is directly part of—modifying its normal protective functions against heart failure, myocardial infarction, hypertension, lung disease, and diabetes mellitus [26,27,28,29]. In fact, ACE2 under normal conditions is responsible for converting angiotensin (Ang) II, produced from Ang I by ACE, into Ang-(1-7), which limits Ang II effects via Ang II type 1 receptors (AT1R), thus preventing an inflammatory and fibrotic milieu. When SARS-CoV-2 binds ACE2, it determines its negative regulation, resulting in Ang II accumulation, responsible for vasoconstriction, profibrotic, and proinflammatory effects, as well as inflammation and tissue fibrosis [30].

Moreover, SARS-CoV-2 infection is associated with severe and diffuse immune system dysregulation.

A pivotal role in immunological disruption is exerted by the priming and activation of the NOD-like receptor protein 3 (NLRP3) inflammasome in host cells, which is able to form pores in cell membranes and consequently release cytosolic content, which leads to inflammatory and lytic programmed cell death, called pyroptosis [31,32,33]. 

In COVID-19, it is induced by a multimodal activation of NLRP3 inflammasome: hyperactivation of Ang II-AT1R axis secondary to ACE2 binding to S protein; direct interaction between SARS-CoV-2 nucleocapsid (N) protein and NLRP3 inflammasome; calcium leakage into the cytosol through ion channels formed by SARS-CoV-2 envelope (E); potassium efflux; and reactive oxygen species (ROS) production induced by SARS-CoV-2 [34,35,36,37,38,39]. All these triggers increase NLRP3 inflammasome activity, which can be the culprit of induction and perpetuation of “cytokine storm” in SARS-CoV-2 patients [40,41].

The “cytokine storm” or “cytokine storm syndrome (CSS)”, observed in many viral and inflammatory diseases and also reported in COVID-19 patients, consists of a lethal inflammatory state with overproduction of cytokines that leads to a hyperinflammatory condition causing severe organ damage, as lung, brain, heart, kidney and skin injury [42,43,44,45,46]. Many studies have confirmed that COVID-19 patients have elevated interleukin plasma concentrations and that cytokine serum levels correlate with the disease severity [47,48]. At the same time, there is evidence that disease severity, inflammation level, and organ failure are associated with elevated endothelial activation and disruption markers. Magro et al. showed significant complement activation resulting in damage to microvascular endothelial cells and subsequent activation of the coagulation pathway [14]; moreover, another study by Ruhl et al. reported extended endothelial activation and disruption, broad dysregulation of fibrinolysis and coagulation, and elevated proinflammatory cytokines in intensive care unit patients with the highest COVID-19 severity [49].

Another mechanism involved in COVID-related endotheliopathy is oxidative stress, resulting from the overproduction of ROS, which reduces vessel relaxation and exacerbates vessel contraction through reduced bioavailability of vasodilator molecules, in particular nitric oxide (NO) [2,50,51,52,53,54]. Increased ROS levels are responsible for proinflammatory cytokine production and secretion and stimulate the expression of adhesion molecules, enhancing leukocyte adherence and extravasation into the vascular wall [55,56,57,58]. In this way, oxidative stress contributes to creating a proinflammatory milieu with a vasoconstriction effect but can play a role also in remodeling the microvascular environment [59].

In COVID-19 patients, in addition to cytokine overproduction and secretion, it has been observed that NO deficiency is linked to a weakened capability of inhibiting the viral replication cycle [60,61]. Moreover, it has been shown that downregulation of ACE2 after binding with SARS-CoV-2 and consequent overexpression of Ang II-AT1R axis determine an overactivation of NADPH-oxidase (Nox) 2, one of the most important cellular producers of ROS, with proinflammatory and vasoconstriction activity [62,63].

An important structure involved in endothelial dysfunction is represented by the endothelial glycocalyx (EG) [64]. The EG is a carbohydrate-rich layer lining the endothelium, covering the surface of blood vessels, and made of soluble plasma proteins which are linked to each other directly or via proteoglycans. The negatively charged EG has the function of a barrier, repelling blood cells, regulating capillary permeability, preventing leukocytes binding and consequently adhesion, and has anticoagulant action [65,66,67,68,69,70,71,72]. Several studies show that the EG is damaged in severe COVID-19 patients, and the increased plasma levels of glycocalyx components were associated with an increased risk for COVID-19 severity and mortality [64,73,74,75,76]. Several drugs that protect EG from damage are already used in COVID-19, such as heparin and tocilizumab [77,78,79]. 

Finally, emerging interest is referred to endothelial extracellular vesicles (EVs), which are considered interlocutors in the crosstalk between inflammation and coagulation set up by SARS-CoV-2 infection. A consequence of the endothelium activation is represented by the release of endothelial cells and EVs into the bloodstream [2,80,81]. Thus, EVs originating from the endothelium are currently deemed important in the progression of CV disease and represent a promising biomarker of endothelial damage [82]. The role of EVs in COVID-19 needs to be further clarified. However, studies showed that EVs expressing CD62 (E-selectin) in COVID-19 patients were related to the severity of patients at admission and to in-hospital mortality and that EVs expressing endothelial tissue factor (TF) have a central role in activating the coagulation pathways. A rise in plasma TF levels has been associated with an increased risk of clinically relevant thromboembolic events up to 28 days after [83,84].

## 3. Long COVID

### 3.1. Epidemiology and Risk Factors

A significant proportion of patients report a wide range of persistent symptoms—mainly fatigue, dyspnea or shortness of breath, myalgias, and sleep disturbances—that do not resolve over the course of many weeks after acute SARS-CoV-2 infection and are not justified by an alternative diagnosis. This condition identifies the so-called “long COVID syndrome” or “post-acute sequelae of COVID-19 (PASC)” [85]. In the United Kingdom National Institute for Health and Care Excellence (NICE) guidelines, long COVID has been defined as the persistence of symptoms beyond 4 weeks from SARS-CoV-2 infection. This term encompasses two phases: ongoing symptomatic phase (4–12 weeks), called post-acute-COVID, and post-COVID-19 syndrome (>12 weeks), called long COVID, based on the duration of symptoms [8]. World Health Organizations (WHO) defines long COVID as the persistence or development of new symptoms 12 weeks after the initial SARS-CoV-2 infection. Symptoms should last more than 8 weeks, after having excluded other causes [86].

At least 75 million subjects have developed long COVID, an estimated incidence of 10% of infected people and more than 766 million documented COVID-19 cases worldwide [87].

It is widely known that there is a strong link between acute COVID-19 severity and the incidence of long COVID syndrome, even if long COVID has been diagnosed also in patients who experienced only mild or asymptomatic infection [88]. In particular, several studies have assessed that hospitalized patients have typically reported a higher estimated prevalence of symptoms (e.g., 76% in Huang et al. [89], 71% in Evans et al. [90]) when compared with community studies (e.g., Sudre et al. [91]), thus reflecting the complex relationship between the severity of acute illness, higher burden of comorbidities, and persistent symptoms [92].

The risk of developing PASC or long COVID syndrome can be estimated using several scores, such as the PASC score, a clinical symptom-based score combined with an antibody signature [91,93,94].

Moreover, several pieces of evidence show that some conditions, such as aging, asthma, obesity, poor pre-pandemic general health, and female sex, seem to be associated with the risk of developing long COVID syndrome [91]. This association seems to be likely for patients with severe COVID-19 but not for mild and non-hospitalized cases [95]. About poor general health before infection, a special mention goes to cancer patients. A retrospective multicenter registry study, OnCovid, showed that 15% of cancer survivors of SARS-CoV-2 infection present, at their first oncological follow-up, sequelae of viral infection [96]. Up to 25% of patients report symptoms for more than 6 months after resolution of infection, with evidence of psychophysical impairment requiring medical treatment in 14% of patients [97]. This suggests that, in patients with cancer, organ damage, of which symptomatic sequelae are a proxy, could contribute to a significant worsening of patient survival, regardless of oncological prognosis.

Another variable associated with the risk of long COVID seems to be virus variant: a recent population study found that individuals infected with the SARS-CoV-2 Omicron variant had a lower risk of having post-COVID complaints after 90 days after testing positive than individuals infected with the Delta variant [98].

The development of COVID-19 vaccines has significantly reduced the risk of developing long COVID syndrome: a matched-cohort study found a remarkable decrease in the incidence of long COVID among vaccinated subjects: those receiving two doses two weeks before contracting COVID-19 had a 41% lower odds of long COVID, when compared to unvaccinated patients [99].

In this regard, as of October 2022, the Robert Koch Institute (RKI) recommends COVID vaccination for all women who currently wish to have children and to those who are already pregnant [100]. This because serious pregnancy complications (e.g., hypertensive disorders, postpartum bleeding) occurred 40% more frequently in women infected with SARS-CoV-2 than in seronegative women [101]. A Brazilian case-control study of 84 pregnant women with COVID-19 confirmed by PCR found that 80% of study participants suffered from long-term COVID symptoms, and one-third of women had persistent symptoms after childbirth [102]. Unfortunately, the research situation on long COVID in pregnancy and lactation is still at the beginning, and the literature is limited and there is not much epidemiological data available.

### 3.2. General and Endothelial Pathogenetic Mechanisms

It is likely that patients with long COVID have different underlying biological factors driving their symptoms, none of which are mutually exclusive: immune dysregulation with autoimmune response, stimulation of clotting cascades and related thrombo-inflammation, dysregulation of the RAAS, endothelial cell damage, persisting reservoirs of SARS-CoV-2 in tissues, and chronic hypoxia [11,88,103,104,105].

The persistence of the proinflammatory mechanisms participating in the acute phase of COVID-19 may also determine immune alteration in the long COVID phase. The most remarkable alterations have been described in T-lymphocyte function (exhausted T cells), B- and T-lymphocyte number (reduction in CD4+ and CD8+ cells, reduction in naïve T and B cells), and in an increased expression of PD-1 and type I and type III interferon [104,106]. In this light, SARS-CoV2 could be associated to the host immune response dysregulation during the acute disease that seems to persist also in the post-acute phase allowing reactivation and reinfection by other previously contracted pathogens, thus driving chronic symptoms [88]. As in the acute phase, in long COVID patients, elevated levels of cytokines, particularly IL-1b, IL-6, and TNF, have been found [107,108]. Finally, another important finding in long COVID is the presence of elevated levels of autoantibodies responsible for an autoimmune response that targets different tissues, organs, or system immunomodulatory proteins [109,110]. The precise mechanisms by which SARS-CoV-2 infection triggers autoantibody production remain unknown, but several studies showed the presence of autoantibodies against G-protein-coupled receptors (GPCRs) and RAAS-related molecules. Antibodies directed against Ang-II interfere with signaling between Ang-II and its AT1 and ACE2 receptors, correlating with enhanced pro-inflammatory responses and increased disease severity. In fact, patients with moderate and severe disease have higher autoantibody levels than healthy controls and those with mild COVID-19 disease [111,112].

Moreover, it is possible that, at least in some patients, SARS-CoV-2 may drive symptoms by persisting in certain body sites or tissue reservoirs: studies have demonstrated that some infected patients with SARS-CoV-2 do not successfully clear the virus over a long period [113]. As previously mentioned, SARS-CoV-2 has the potential to leave cells as small secretory vesicles that then release virus [114]. In long COVID, SARS-CoV-2 may hide in these extracellular vesicles (EVs) and re-attach various tissues and organs through the circulatory system [80,83,114,115,116].

Immune dysregulation coupled with the persistent viral reservoirs may be responsible for a chronic inflammatory response, and, consequently, endothelial damage is inevitable [80].

A condition of chronic hypoxia has been demonstrated in long COVID patients, probably due to the increased production of ROS and the consequent loss of the protective effect of NO in addition to the increase in AngII levels and the proinflammatory state triggered by the virus. Notably, hypoxia also activates the production of NF-κB, the master switch for the transcription of genes that elicit inflammatory responses [117]. The interplay between the infection and the inflammation related to hypoxia worsens endothelial function and accelerates both inflammation and tissue damage.

Chronic hypoxia involves pulmonary structural changes (hypertension, embolism, and fibrosis), vascular changes, decreased lung function, and long-term changes in the size and stiffness of blood cells [85,118,119,120]. Hypoxia also provides conditions under which immune cells produce more inflammatory cytokines [121,122].

Ultimately, prolonged viral presence, chronic hypoxia, and persistent inflammatory response contribute to persistent endothelial damage, with prolonged coagulation activation, microvascular injury, sustained (low-grade) clot formation in damaged vasculature, and thrombosis, driving systemic damage in patients [80,105,123].

Different endothelial biomarkers and tests for vascular function have been proposed for endothelial dysfunction evaluation in patients with long COVID [7,116,124].

Studies have been conducted to understand the potentially detrimental effects of SARS-CoV-2 on systemic vasculature, with evidence of impaired NO availability and lower vascular function, as shown by reduced reactive hyperemia index (RHI) and reduced flow-mediated dilation (FMD) several weeks after testing positive for SARS-CoV-2 [124,125,126].

Recently, in a prospective observational study, our group showed that endothelial dysfunction, measured as FMD reduction three months after the acute phase, had a linear relationship with acute COVID-19 severity, independent of other typical risk factors for endothelial dysfunction [126]. Moreover, FMD continues to correlate to COVID-19 severity also after 3 months, unlike CRP measurement. The hypothesis is that patients with moderate/severe COVID-19 have a local sub-inflammatory status that could remain localized in the vasculature, responsible for persisting endothelial dysfunction in the middle-term, and not inducing systemic production of CRP.

FMD may be a surrogate marker of persistent endothelial inflammation in subjects with previous COVID-19 and potentially allow to identify subjects with higher risk of post-acute COVID-19 syndrome [126].

An interesting study of long COVID by Chioh et al. has found another possible biomarker of vascular injury in circulating endothelial cells (CECs). They have shown elevated levels of CECs, dislodged from damaged blood vessels, in patients who recovered after COVID-19 infection compared to healthy controls [126,127].

On the other hand, as demonstrated by epidemiological data, people with diabetes, hypertension, heart failure, coronary heart disease, smoking, and advanced age, which correspond to a pre-existing high risk of endothelial dysfunction, could be more at risk of developing severe forms of COVID-19 [16,128,129,130,131]. In these populations, COVID-19-related inflammation represents another potential endothelial damage; in fact, it has been shown that these patients had a markedly reduced FMD as a sum of their pre-existing endothelial dysfunction and COVID-related inflammation [126]. These findings all suggest that endothelial dysfunction due to CV disease exacerbated by COVID-19 infection leads to a more severe acute disease, which in turn correlates with a higher prevalence of long COVID. The latter could become a persistent additional factor of endothelial dysfunction, potentially responsible for developing clinically more relevant macro- and microvascular thrombotic events. However, more studies are needed to investigate the burden of such endothelial dysfunction over a longer period.

### 3.3. Clinical Manifestations of Long COVID

**Figure 2:**
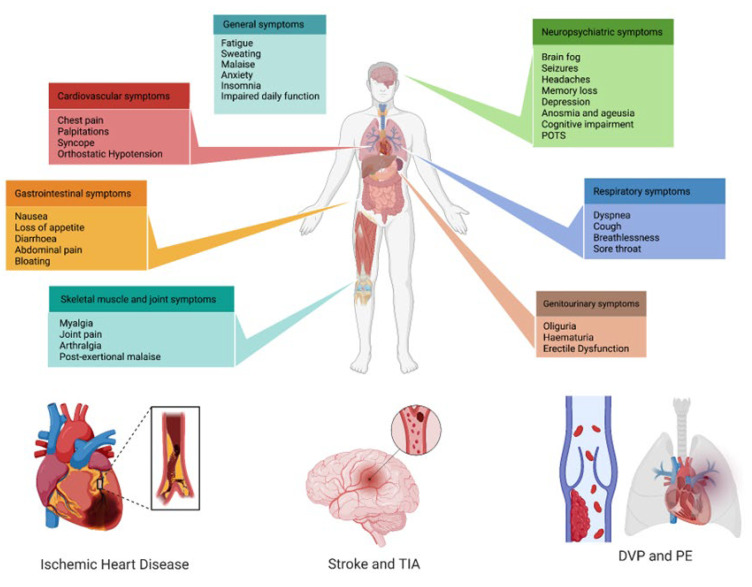
Multi-system clinical presentations of long COVID syndrome, with a special focus on the main cardiovascular implications. POTS: post-orthostatic tachycardia syndrome; TIA: transient ischemic attack; DVP: deep-vein thrombosis; PE: pulmonary embolism.

Nowadays, three years after the COVID-19 pandemic started, the symptoms that patients with long COVID refer to are mostly mild, non-specific, and reversible, but moderate, severe, and persistent symptoms have also been reported [89,132,133]. It has been suggested that three main groups are organically involved in long COVID: neurologic-neuropsychiatric, followed by pneumological, and third, CV symptoms (Figure 2).

Neurological involvement, anosmia, ageusia, cognitive impairments, depression, and anxiety are common features of long COVID [134]. Prominent among these long-term sequelae are persistent cognitive symptoms, identifying the so-called COVID-19 “brain fog,” characterized by impaired attention, concentration, memory, speed of information processing, and executive function [135]. Also, the neuro-vegetative system is involved, as shown by the high prevalence of postural orthostatic tachycardia syndrome [136].

Regarding pulmonary involvement, the most frequently examined symptom was shortness of breath or dyspnea, followed by post-activity polypnea, cough, chest distress, and chest pain, also assessed in children and young adults [137,138]. Approximately half of patients recovering from COVID-19 report chronic dyspnea 2–3 months after infection [85]. The possible underlying mechanism is pulmonary fibrosis and other patterns of lung damage, as shown in persistent radiological and pulmonary functional changes after the acute phase, such as forced vital capacity, forced expiratory volume in the first second, diffusion of carbon monoxide in the lung, 6-min walk test, and end-exercise oxygen saturation [139,140,141,142,143]. In addition to these structural changes, endothelial dysfunction may also have a role in impaired pulmonary functional parameters [144].

Third, CV manifestations are extremely heterogeneous, spanning from venous thromboembolism to ischemic heart and cerebrovascular disease to dysrhythmias, as discussed below.

## 4. Cardiovascular Involvement in Long COVID

As shown also in other viral respiratory infections, CV complications make a significant contribution to morbidity and mortality, as chronic CV disease may become unstable in the setting of an acute viral infection: myocarditis and consequent heart function impairment, coronary plaque rupture secondary to systemic inflammation (stent), and thrombosis due to systemic procoagulant effects [145,146]. 

In addition to acute CV complications, data from the cohort of the US Department of Veterans Affairs national healthcare database (Xie et al.) have shown an increased risk and burden of CV disorders beyond the acute phase of COVID-19, in particular, heart failure, dysrhythmias, ischemic heart disease, and stroke 1 year after SARS-CoV-2 infection, independently of initial severity [9]. 

The well-known association between SARS-CoV-2 transmission and stroke, as well as myocardial infarction risk, suggests a link between impaired blood flow and acute CV risk [124,147,148]. 

Several putative mechanisms are involved in the development of acute and chronic CV complications [146]. The damage mediated by chronic inflammation could be worsened the presence of viral reservoirs in several organs, including the heart, that persist after COVID-19. The acute disease could be worsened by activated inflammatory pathways that could worsen the function of the endothelium by increased production of ROS and nitric oxide synthetase (eNOS) uncoupling [11,149]. Thus, cardiac tissue inflammation persists and evolves in myocardial fibrosis. This could lead to several manifestations of cardiac disease, ranging from reduced ventricular compliance due to an increased stiffness to a reduced contractility due to an impaired myocardial perfusion, to the generation of an arrhythmogenic substrate due to inflammation, fibrosis, and hypoperfusion [92].

Another suggested mechanism that has been evaluated in long COVID CV manifestations is an autoimmune response to cardiac antigens through molecular mimicry; actually, some studies detected the presence of autoantibodies to cholinergic and adrenergic receptors, but more investigations are needed [10,150].

### 4.1. Venous Thromboembolism

Venous thromboembolism is the most considerable sequela of long COVID due to a persistent procoagulant status enduring after the acute phase of SARS-CoV-2 infection, although specific pathogenetic mechanisms are still poorly understood [80,151,152].

Some autopsy studies on patients with acute COVID-19 have demonstrated the presence of disseminated pulmonary (micro) thrombosis throughout the pulmonary vasculature—mostly in severe disease—suggesting its formation directly within the lungs rather than being embolic in origin [14,151,153,154,155].

A hypercoagulable and hypofibrinolytic status has been described in patients 4 months after acute COVID-19, associated with persistent increased levels of D-dimer, factor VIII, plasminogen activator inhibitor-1 (PAI-1) and von Willebrand factor (vWF), markers of endothelial activation. Moreover, COVID-19 might be related to the acquired natural anticoagulant deficiency. Wójcik et al. showed that SARS-CoV2 patients had 17% decreased protein C activity levels, 22% lower free protein S, and a higher prevalence of positive results for anticardiolipin IgM antibodies, as well as a 151% increase in vWF and DDimer (55%) [156]. Mechanisms underlying these persistent anomalies are unclear but may involve sustained endothelial activation [123,157].

Several studies tried to quantify the incidence of ongoing thrombosis risk in patients after discharge, but most results are still limited to small sample sizes and short observation periods. Giannis et al. conducted one of the largest-scale statistical analyses of major thromboembolic events in postdischarge COVID patients. The results showed a higher frequency of thrombotic events in a 90-day period after infection than thought, with 1.55% of patients diagnosed with venous thromboembolism (VTE), 0.9% with deep vein thrombosis (DVT) and 0.85% pulmonary embolism (PE) [158].

Other observational studies have found smaller incidence of these events, as Engelen et al., who found only 0.7% VTE and 0.7% DVT in patients after 6 weeks from acute infection [159]. The incidence of both arterial and venous thrombotic events one month after hospital discharge has been estimated at 2.5%. In the same time-frame, venous thromboembolic disease affected 0.6% of the patients [160]. According to what has been expressed before, these events appeared to be more frequent in people with certain risk factors as age >75 years, CV risk factors (personal history of VTE, ischemic heart disease, carotid or peripheral arterial disease), chronic kidney disease (CKD), IMPROVE-DD VTE score ≥4, and intensive care unit stay [158]. 

Although thrombosis risk gradually increases with disease progression and severity and early anticoagulation has shown beneficial results in hospitalized patients with-COVID-19, there is no sufficient evidence to consider post-COVID patients at high risk of thrombosis, and, consequently, there is no recommendation about the use of anticoagulants after discharge [161,162,163].

Some studies showed that extended thromboembolic prophylaxis beyond hospital discharge might be beneficial, also with a small reduction of mortality from VTE, but it is limited to high-risk patients with an increased risk of thromboembolism from COVID-19; however, evidence is low [159,164].

This issue is still an object of debate, with divergence in different guidelines: CHEST guidelines recommended thromboprophylaxis only during hospitalizations for COVID-19, not suggesting an extended treatment after hospital discharge. Other clinical guidance suggests that extended post-discharge prophylaxis should be considered by balancing the risks of thrombosis and bleeding [165,166,167,168].

### 4.2. Cardio- and Cerebrovascular Diseases, Myopericarditis, and Dysrhythmias

To date, there are insufficient data about the incidence and prevalence of cerebrovascular diseases, ischemic heart disease, myopericarditis, and dysrhythmias in long COVID patients.

Xie et al. analyzed to-date the largest database from the US Department of Veterans Affairs, examining groups by care setting of acute infection (non-hospitalized, hospitalized, and admitted to intensive care) and matching them with contemporary and historical control groups. Composite outcomes of this study consisted of cerebrovascular diseases, dysrhythmias, inflammatory heart diseases, ischemic heart disease, other cardiac disorders, thrombotic disorders, MACEs and any cardiovascular outcomes. They showed that people with COVID-19 had increased risks and 12-month burdens of incident CV disorders 30 days after infection, regardless of other typical CV risk factors, and even in people with a low risk of CV disease before COVID-19 exposure [9].

A direct relationship has been demonstrated between acute infection severity and increasing burden of CV outcomes; interestingly, this increase was observed also in non-hospitalized COVID-19 patients—the majority of people with COVID-19—concluding that the increased post-acute COVID-19 CV outcomes would be attributable sequelae to COVID-19 itself.

People who survived the first 30 days of COVID-19 exhibited an increased risk of stroke and transient ischemic attacks (TIA) [169]. Their etiology has been considered multifactorial, with an important role of thromboembolic events characteristic of the disease [142]. The risks and burdens of a composite of these cerebrovascular outcomes were 1.53 and 5.48 [9].

A higher prevalence has been demonstrated for acute coronary disease, myocardial infarction, ischemic cardiomyopathy, and angina [9]. The potential etiologies could be the perpetuation of inflammatory damage, stress-induced cardiomyopathy, and cytokine release. For COVID patients 90 days following discharge, Giannis et al. documented a frequency of arterial thromboembolic events of 1.71%, including stroke, myocardial infarction, major adverse limb event, and systemic embolism [158].

Crook et al., in a cohort study, showed that long COVID patients two months after infection often had chest pain associated with a persistent mild cardiac troponin elevation, frequently sustained from persistent myocardial inflammation [170].

Several studies underlined the presence of modifications in surface electrocardiogram or 24 h electrocardiographic monitoring in long COVID, and recent works underlined that they can be temporary or may persist indefinitely. The prevalence of such alterations is highly variable, ranging from less than 1% in young athletes to more than 27.5% in elderly, comorbid patients who required hospitalization [171]. Common surface electrocardiographic changes include sinus tachycardia, ST segment abnormalities (both aspecific and elevation), morphological alterations of T-wave, long QT interval, reduced QRS voltages, and new onset of bundle branch blocks [132]. Alterations of sinus rhythm are common after COVID-19 and consist of phases of sinus tachycardia or bradycardia [172,173].

## 5. Clinical Insights and Future Directions

During 2019–2021, the COVID-19 pandemic was the most important healthcare emergency of the last decades; actually, epidemiological data denote long COVID as a more relevant issue, although emerging evidence indicates an increasing number of patients will suffer from long COVID in the future. The disease burden associated with long COVID appears durable, long-term effects remain unknown, and our current knowledge of pathophysiology remains limited. It is very likely that the long-term effects of COVID-19, primarily exerted on the endothelial cells, may be responsible for an increase in CV risk but also for other organ manifestations, which underscores the need for multispecialty input.

This suggests the importance of ongoing systematic and, in particular, CV surveillance of long COVID patients to establish epidemiology of middle- and long-term complications. Moreover, while the risk of long COVID can be easily assessed for patients with severe COVID-19, it could reasonable to assess multi-organ screening, especially CV investigations (including FMD determination), in particular for mild and non-hospitalized cases, in order to reveal hidden systemic diseases and the presence of risk factors. Moreover, the lack of data underlines the need for further studies to elucidate the role of endothelium as a game-changer and to better understand metabolic pathways, also involving the pre-incubated endothelium, as possible targets of therapeutic interventions in order to reduce cytokine overproduction. For this reason, vaccination before COVID-19 infection remains the only instrument to prevent the occurrence and the severity of symptoms in long COVID patients after infection.
